# PTIP promotes recurrence and metastasis of hepatocellular carcinoma by regulating epithelial-mesenchymal transition

**DOI:** 10.18632/oncotarget.16436

**Published:** 2017-03-22

**Authors:** Shusheng Leng, Mingyang Yang, Yanhua Zhao, Jingfeng Zhao, Zhijun Zeng, Yunpeng Yang, Jiatian Yuan, Bo Lv, Fan Jun, Bing Wang

**Affiliations:** ^1^ General Surgery Department, Affiliated Hospital/Clinical Medical College of Chengdu University, Chengdu 610081, China; ^2^ Intensive Care Unit, The First People's Hospital of Chengdu (Chengdu Combine Traditional Chinese and Western Medicine Hospital), Chengdu 610041, China; ^3^ Department of Laboratory Medicine/Clinical Research Center of Laboratory Medicine, West China Hospital of Sichuan University, Chengdu 610041, China; ^4^ General Surgery Department, Chongqing Dazu District People's Hospital, Chongqing 402360, China; ^5^ Department of Geratic Surgery, Xiangya Hospital, Central South University, Changsha 410008, China; ^6^ Department of Pathology, Affiliated Hospital/Clinical Medical College of Chengdu University, Chengdu 610081, China

**Keywords:** PAX interacting protein1 (PTIP), hepatocelluar carcinoma, miRNA, epithelial-mesenchymal transition (EMT), metastasis

## Abstract

Hepatocellular carcinoma (HCC) is one of the most lethal tumors worldwide, which is mainly due to the high recurrence and metastasis rate after hepatectomy. In this study, we found that PTIP expression was dramatically upregulated in human HCC tissues and cell lines. High expression of PTIP was shown to be associated with aggressive clinicopathological features, including liver cirrhosis, vascular invasion and advanced stage. In addition, PTIP overexpression was independently associated with shorter survival and increased HCC recurrence in patients. Knockdown of the PTIP expression significantly inhibited invasion and metastasis *in vitro* and *in vivo*, whereas ectopic expression of PTIP significantly promoted invasion and metastasis. Mechanistically, PTIP promotes HCC progress by facilitating epithelial-mesenchymal transition (EMT). Notably, we also found that PTIP might increase miR-374a expression to promote EMT and metastasis in HCC. In summary, our study identified PTIP as a new potential prognostic indicator and therapeutic target for HCC.

## INTRODUCTION

Hepatocellular carcinoma (HCC) is one of the most commonly diagnosed cancers and the leading cause of cancer-related deaths worldwide. Approximately 782,500 new cases and 745,500 deaths occurred worldwide in 2012 [1]. It is estimated that the number of new liver cancer cases and deaths was 343700 for 2015 in China, which accounts for almost half of the total number of HCC [2]. Although treatments for HCC have been greatly improved, the overall survival of patients with HCC remains unsatisfactory. It is mainly due to the high recurrence and metastasis after hepatectomy [3, 4]. Therefore, it is important to further dissect the mechanisms underlying metastasis of HCC, which would be of great value for developing effective prognostic and therapeutic strategies.

PTIP, PAX transactivation domain-interacting protein, also known as Paxip1, is a nuclear-localized protein with functions in DNA repair, embryonic vascular development and embryonic stem cell pluripotency [5–8]. It is generally accepted that PTIP regulates the deposition of H3k4me and gene expression by recruiting the MLL3/MLL4 methyltransferase complex to gene-specific promoters or enhancers [9–11]. High PTIP expression is associated with increased progression-free survival in Brca2-associated ovarian cancers [12]. The loss of PTIP might confer genome stability to Brca-deficient cells, and protects Brca-deficient cells from DNA damage and cell death [12]. However, the biological function and clinical significance of PTIP in HCC remains largely unknown.

In this study, we started from analyzing the expression characteristics of PTIP in tumor tissues and cell lines. The biological function and the underlying molecular mechanisms of PTIP in HCC metastasis were also investigated *in vitro* and *in vivo*. Our results determined the clinical significance of PTIP as a prognostic marker and potential therapeutic target in human HCC tissues as well as cell lines.

## RESULTS

### Expression of PTIP was prominently up-regulated in HCC samples and cell lines

**Figure 1:**
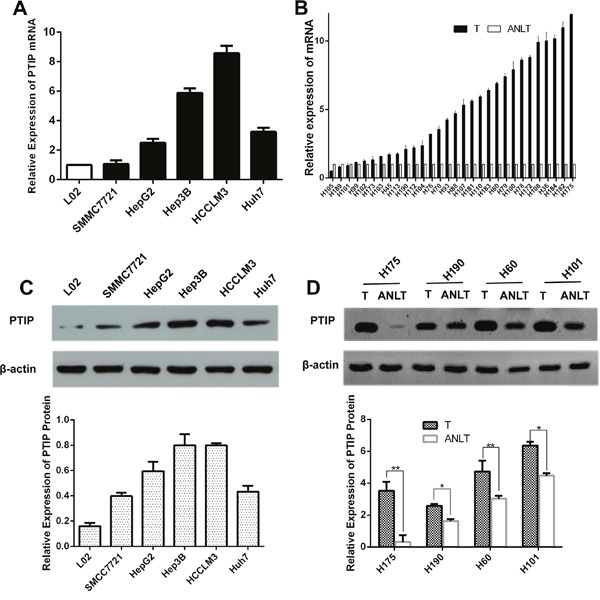
PTIP expression is up-regulated in HCC tissues and cells **(A)** PTIP mRNA of human HCC cell lines was up-regulated by quantitative real-time PCR (qRT-PCR). **(B)** PTIP mRNA expression in 30 pairs of HCC tissues was higher than their corresponding adjacent nontumorous liver tissues (ANLTs). Expression level of PTIP was determined by qRT-PCR and normalized to GAPDH. Fold change was analyzed using the formula 2^-(ΔΔCT[HCC/ANLT])^. **(C)** PTIP protein expression in HCC cell lines and normal liver cell line L02 was detected by western blot. Below was the statistical analysis of results. The representative western blot results showed that Hep3B and HCCLM3 cells had the highest PTIP expression level compared to L02 cells. **(D)** PTIP protein in paired HCC tissues was significantly higher than those in ANLTs. Below was the statistical analysis of results. GAPDH and β-actin was used as an internal loading control.

To investigate the PTIP expression levels in HCC tissues and cells, quantitative real time-PCR (qRT-PCR) and western blotting were performed. Compared with the normal immortalized human liver cells L02, PTIP mRNA was highly expressed in HCC cells (Figure 1A). Thirty pairs of HCC tissues and corresponding adjacent non-tumor liver tissues (ANLTs) were analyzed with qRT-PCR, and the results showed that PTIP expression in HCC tissues was markedly higher than that of ANLTs. The median fold-change was 4.46 (range, 0.50-11.93) (Figure 1B). Moreover, PTIP protein level in cell lines and tissues was also determined by western blotting. Consistent with the mRNA expression level, western blotting results showed that the expression of PTIP protein in HCC cells was significantly higher than that of L02 (Figure 1C). PTIP protein was up-regulated in HCC tissues compared with that in ANLTs (Figure 1D). ELISA was developed to evaluate PTIP in ten serum samples. It was a pity that PTIP could not detected in serum.

### High PTIP expression was correlated with poor clinicopathologic characteristics and prognosis of HCC

**Figure 2:**
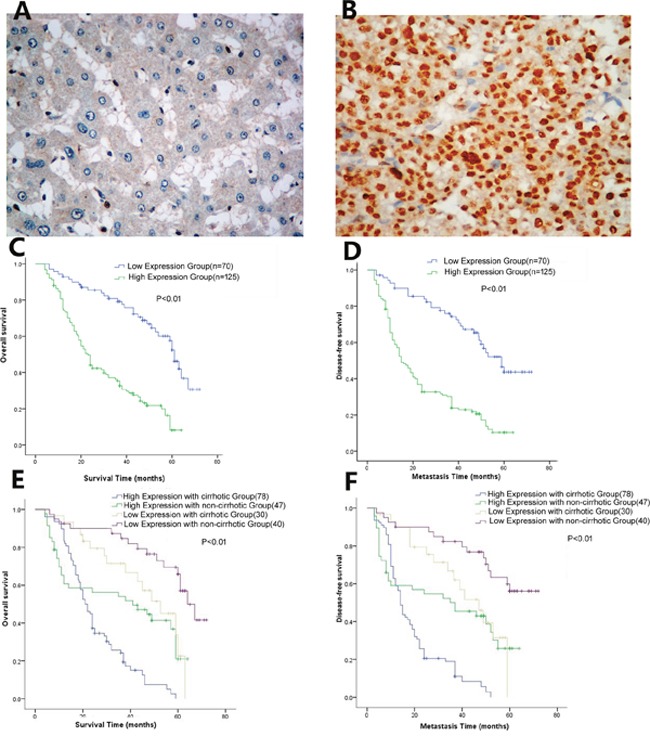
Immunohistochemistry analysis of PTIP protein expression in HCC tissues **(A)** Representative IHC images of PTIP expression in ANLT. **(B)** Representative IHC images of PTIP in HCC. **(C)** The overall survival time of PTIP high expression group was significantly poorer compared to PTIP low expression group (P<0.01). **(D)** PTIP high expression group also had poorer disease-free survival than PTIP low expression group (P<0.001). **(E, F)** The association between PTIP expression and OS/DFS in non-cirrhotic versus cirrhotic patients. The results showed that high PTIP expression with cirrhotic had significantly lower OS and DFS time than low PTIP expression without cirrhotic group (P<0.01)

Immunohistochemistry (IHC) analysis of PTIP expression in HCC tissues revealed that PTIP protein was predominantly located in the nucleus, and the expression of PTIP was significantly higher in HCC tumors than that in ANLTs (Figure 2A, 2B). According to the immunohistochemistry results, we divided the patients into two groups: high expression group (n=125), low expression group (n=70).

To examine the relationship between PTIP expression and clinicopathological features, we analyzed their correlations. The results showed that PTIP expression was positively correlated with liver cirrhosis (*P*=0.008), tumor number (*P*=0.029), vascular invasion (*P*= 0.003), capsular formation (*P*<0.001), Edmondson-Steiner grade (*P*=0.010), BCLC Stage (*P*=0.018) (Table 1). Furthermore, we analyzed the relationship between PTIP expression and prognosis. The tumor number (Hazard ratios (HR): 2.516 *P*=0.010), vascular invasion (HR: 4.154, *P*=0.026), liver cirrhosis (HR: 4.038, *P*=0.018), Edmondson-Steiner grade (HR: 3.010, *P*=0.031) and PTIP expression (HR: 4.980, *P*=0.014) were recognized as independent risk factors for overall survival (OS) by both the univariate analysis and subsequent multivariate survival analysis (Table 2). Additionally, the tumor number (HR: 2.273, *P*=0.016), vascular invasion (HR: 5.177, *P*=0.017) and PTIP expression (HR: 5.112; *P*=0.018) were verified as independent risk factors for disease-free survival (DFS) by both univariate and multivariate survival analysis (Table 3).

**Table 1:** Correlations between PTIP and clinicopathologic variables of HCC

		PTIP		
Clinicopathologic variable	No.	Low	High	*P* value
**Gender**				
Female	51	16	35	0.433
Male	144	54	90	
**Age (years)**				
≤50	104	42	62	0.163
>50	91	28	63	
**HBsAg**				
Positive	136	46	90	0.359
Negative	59	24	35	
**Liver cirrhosis**				
Presence	108	30	78	**0.008**
Absence	87	40	47	
**AFP**				
**≥400μg/L**	124	44	80	0.874
**< 400μg/L**	71	26	45	
**Tumor number**				
Solitary	90	25	65	**0.029**
Multiple (≥2)	105	45	60	
**Tumor size**				
≤5 cm	80	35	45	0.057
>5 cm	115	35	80	
**Vascular invasion**				
Presence	89	22	67	**0.003**
Absence	106	48	58	
**Capsular formation**				
Presence	69	42	27	**<0.001**
Absence	126	28	98	
**Child-Pugh**				
**A**	160	59	101	0.543
**B**	35	11	24	
**Edmondson-Steiner grade**				
Low grade (I and II)	102	28	74	**0.010**
High grade (III and IV)	93	42	51	
**BCLC Stage**				
0/A	43	22	21	**0.018**
B/C	152	48	104	

**Table 2:** Univariable and multivariable analysis of overall survival(OS) and PTIP by cox proportional hazards regression model

		Univariable analysis		Multivariable analysis	
Variables	No.	HR (95% CI)	*P* value	HR (95% CI)	*P* value
**Gender**					
Female	51	1			
Male	144	0.620(0.350-1.825)	0.269		NA
**Age (years)**					
≤50	104	1			
>50	91	0.554 (0.421-1.628)	0. 085		NA
**HBsAg**					
Positive	136	1			
Negative	59	0.267(0.126-1.020)	0.440		NA
**AFP**					
**≥400μg/L**	124	1			
**< 400μg/L**	71	3.282(0.866-7.905)	0.859		NA
**Tumor number**					
Solitary	90	1		1	
Multiple (≥2)	105	3.142(2.175-4.432)	**0.002**	2.516 (1.470 - 6.541)	**0.010**
**Tumor size**					
≤5 cm	80	1			
>5 cm	115	1.952(0.590-5.280)	0.218		NA
**Vascular invasion**					
Absent	89	1		1	
Present	106	4.580(2.105-6.832)	**0.012**	3.033 (1. 692-7.541)	**0.026**
**Capsular formation**					
Presence	69	1		1	
Absence	126	2.455(1.230-4.460)	**0.032**	2.254 (0.790 - 9.574)	0.128
**Liver cirrhosis**					
Absent	87	1		1	
Present	108	3.994(1.632-7.244)	**0.008**	4.038(1.508-10.280)	**0 .018**
**Child-Pugh**					
A	160	1			
B	35	0.856(0.356-2.780)	0.839		NA
**Edmondson-Steiner grade**					
Low grade (I and II)	102	1		1	
High grade (III and IV)	93	2.940(1.435-5.788)	**0.020**	3.010(1.438- 14.509)	**0.031**
**BCLC Stage**					
0/A	43	1			
B/C	152	3.847(0.750-9.621)	0.080		NA
**PTIP expression**					
Low	70	1		1	
High	125	4.267(2.524-9.771)	**0.021**	4.980 (2 .016 - 8.052)	**0.014**

**Table 3:** Univariable and multivariable analysis of recurrence-free disease(RFS) and PTIP by cox proportional hazards regression model

		Univariable analysis		Multivariable analysis	
Variables	No.	HR (95% CI)	*P* value	HR (95% CI)	*P* value
**Gender**					
Female	51	1			
Male	144	0.833(0.147-2.422)	0.252		NA
**Age (years)**					
≤50	104	1			
>50	91	0.672 (0.518-1.353)	0.180		NA
**HBsAg**					
Positive	136	1			
Negative	59	0.790(0.309- 1.470)	0.126		NA
**AFP**					
**≥400μg/L**	124	1			
**< 400μg/L**	71	0.894(0.557- 3.824)	0.070		NA
**Tumor number**					
Solitary	90	1		1	
multiple(≥2)	105	2.357(1.264-8.660)	**0.029**	2.273(1.510-6.427)	**0.016**
**Tumor size**					
≤5 cm	80	1			
>5 cm	115	1.513 (0.471-3.069)	0.582		NA
**Capsular formation**					
Presence	69	1		1	
Absence	126	2.581 (1.343-3.039)	**0.046**	2.104(0.884-3.477)	0.242
**Liver cirrhosis**					
Absent	87	1			
Present	108	3.462 (1.625-4.576)	**0.037**	2.708(0.628-4.210)	0.128
**Vascular invasion**					
Absent	87	1		1	
Present	108	5.492 (1.527-9.212)	**0.002**	5.177(1.612-10.210)	**0.017**
**Child-Pugh**					
A	160	1			
B	35	0.684(0.416-3.701)	0.684		NA
**Edmondson-Steiner grade**					
Low grade (I and II)	102	1			
High grade (III and IV)	93	1.810 (0.510-2.854)	0.616		NA
**BCLC Stage**					
A	43	1			
B/C	152	0.548(0.181- 1.868)	0.451		NA
**PTIP expression**					
Low	70	1		1	
High	125	5.213 (1.569-8.867)	**0.027**	5.112(1.872-9.170)	**0.018**

In our study, the proportion of tumor recurrence was 68.2% (133/195) and disease-free survival (DFS) rate was 32.8%(62/195). Kaplan-Maier analysis was exploited to investigate the patient overall survival (OS) and disease free survival (DFS) rates. The survival curves showed that HCC patients with high expression of PTIP had lower OS and DFS time than those with low expression group (Figure 2C, 2D). Meanwhile, 1, 3, 5-year overall survival rate in PTIP high-expression group was significantly lower than that of the PTIP low-expression group (50% vs. 85%;23% vs. 69%;12% vs. 30%, respectively, *P*<0.01), and 1, 3, 5 years disease-free survival rate in PTIP high-expression group was also lower than the low-expression group (37% vs. 85%; 21% vs. 65%; 11% vs. 41%, *P*<0.01). Noteworthy, subgroup analysis showed that high PTIP expression with cirrhotic had significantly lower OS and DFS time than low PTIP expression without cirrhotic group (P<0.01) (Figure 2E, 2F). These results demonstrated that high PTIP expression level was closely correlated with poor survival and could be used as a novel independent prognosis biomarker for HCC patients after hepatic resection.

### PTIP promoted proliferation, invasion and metastasis of HCC *in vitro* and *in vivo*

For further investigating the function of PTIP in HCC invasion and metastasis *in vitro* and *in vivo*, we compared the expression of PTIP in five HCC cell lines (SMCC7721, HepG2, Hep3B, HCCLM3, Huh7). And we selected PTIP low expression cell lines HepG2 and PTIP high expression cell lines HCCLM3 for further study. We employed shRNA method to inhibit the expression of PTIP in HCCLM3 and ectopic expression PTIP in HepG2. The knockdown and overexpression efficiency was confirmed by real time PCR. The results showed that the PTIP-RNAi1 could significantly reduce PTIP expression and the overexpression efficiency was also improved (Supplementary Figure 1A, 1B). Then, we went on functionally characterizing PTIP by focusing on its effect on proliferation and migration of HCC cells.

**Figure 3:**
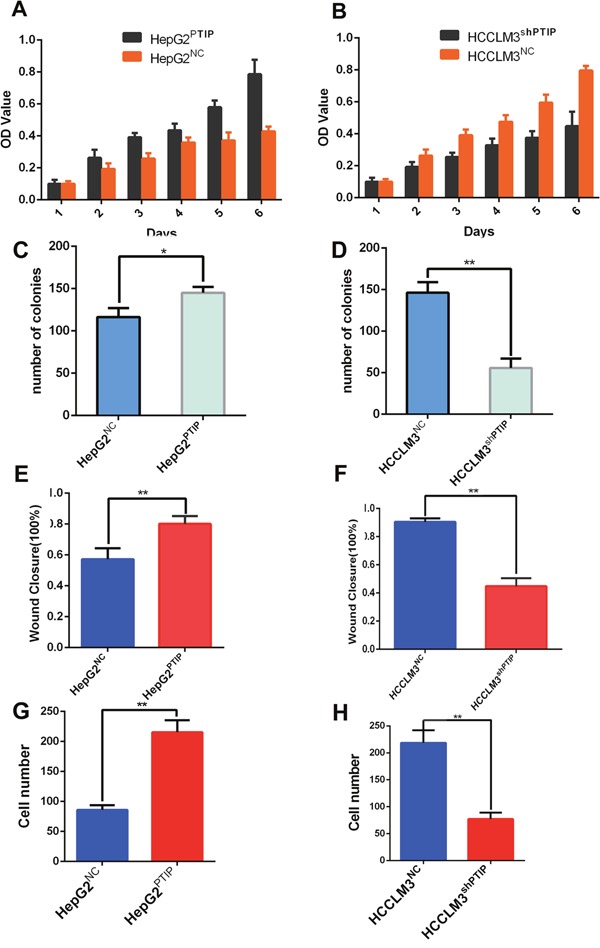
PTIP promotes proliferation and invasion of HCC cells *in vitro* HCC cell lines infected with PTIP expression lentivirus or PTIP inhibition lentivirus or NC were used in these studies. Effect of PTIP on proliferation of HCCLM3 and HepG2 cell lines was assessed using MTT assays and colony formation. **(A, B)** The results of MTT assays showed PTIP promoted cell growth. **(C, D)** Effect of PTIP was on colony formation of HCC cell lines. The colonies >40 um were counted and their numbers were compared. The Wound healing assay and Transwell assay were performed to analyze the effect of PTIP on the motility and invasion of HCC cell lines. The percentage of wound healing or cells passing through the transwell membranes of each well was calculated and is compared in the diagrams. **(E, F)** The results of Wound healing assay showed that PTIP promoted HCC cells migration. **(G, H)** The results of Transwell assay showed that PTIP promoted invasion. Representative images of above diagrams are supplied in Supplementary Figures 1, 2, 3 and 4. *P<0.05; **P<0.01; ***P<0.001.

Methyl thiazol tetrazolium (MTT) assay and colony formation assay were performed to assess the effect of PTIP on cell proliferation. HCCLM3 cells expressing anti-PTIP showed a lower proliferation rate and fewer numbers of colonies than control cells (Figure 3B, 3D and Supplementary Figure 2A). In contrast, HepG2 cells expressing PTIP exhibited higher proliferation rate and greater number of colonies than control cells (Figure 3A, 3C, Supplementary Figure 2B). Furthermore, we employed the wound healing and transwell assays to analyze the function of PTIP in HCC cell invasion and migration. The results showed that HCCLM3^shPTIP^ cells closed much slower and more invasive cells than that of HCCLM3^NC^ cells (Figure 3F, 3H, Supplementary Figures 3A and 4A), whereas HepG2^PTIP^ cells had markedly increased migratory and invasive capacity. (Figure 3E, 3G, Supplementary Figures 3B and 4B). These results revealed that PTIP enhances HCC cells proliferation and invasion potentialities.

**Figure 4:**
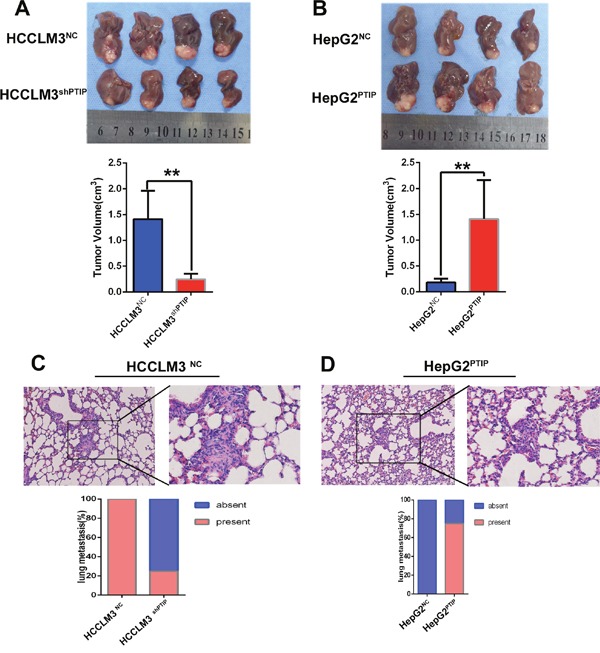
PTIP promotes metastasis of HCC *in vivo* The orthotopic HCC tumors mouse model was constructed by using HCCLM3^NC^, HCCLM3^shPTIP^, HepG2^NC^ and HepG2^PTIP^. Cells (2 × 10^6^) were injected subcutaneously into the left upper flank regions of nude mouse (3-4 weeks of age, male, BALB/c). After one month, the subcutaneous tumor tissues were obtained and cut into commensurate fragments of approximately 1 mm^3^. Then, each fragment was implanted into the liver (four mice for each group). **(A)** The results showed that tumor volumes of HCCLM3^NC^ group was significantly larger than that of HCCLM3^shPTIP^ group(1.41 ± 0.27cm^3^ versus 0.25 ± 0.05 cm^3^, P =0.02). **(B)** The tumor volumes of HepG2^NC^ group was significantly smaller than that of HepG2^PTIP^ group (0.18 ± 0.04cm^3^ versus 1.41 ± 0.38 cm^3^, P =0.02). **(C, D)** Representative pictures of lung metastasis; metastatic nodules proportion or lungs was calculated and compared. (C) The rate of lung metastasis in HCCLM3^NC^ group was higher than that of HCCLM3^shPTIP^ group. (D) The rate of lung metastasis in HepG2^NC^ group was lower than that of HepG2^PTIP^ group. Original magnification: left, 100 ×; right, 400 ×.*P<0.05; **P<0.01 based on the Students test. Error bars, standard deviation.

To verify the above findings *in vivo*, we established orhtotopic xenograft tumor models. We observed that HCCLM3^shPTIP^ cells-derived tumors were smaller and grew slower than HCCLM3^NC^ cells-derived tumors, whereas HepG2^PTIP^ cells-derived tumors were larger and grew more rapidly than HepG2^NC^ cells-derived tumors (Figure 4A, 4B). More importantly, as compared with the HCCLM3^NC^ group, HCCLM3^shPTIP^ group showed a dramatic increase in pulmonary metastasis rate (4/4(100%) vs 1/4(25%). (Figure 4C). However, compared with HepG2^NC^ group, the overexpression of PTIP in HepG2 cells significantly increased the pulmonary metastasis rate (0/4(0%) vs3/4(75%), (Figure 4D). In summary, these results demonstrated that PTIP can enhance the metastatic potential of HCC *in vivo*.

### PTIP facilitated epithelial-mesenchymal transition through miR-374a in HCC

**Figure 5:**
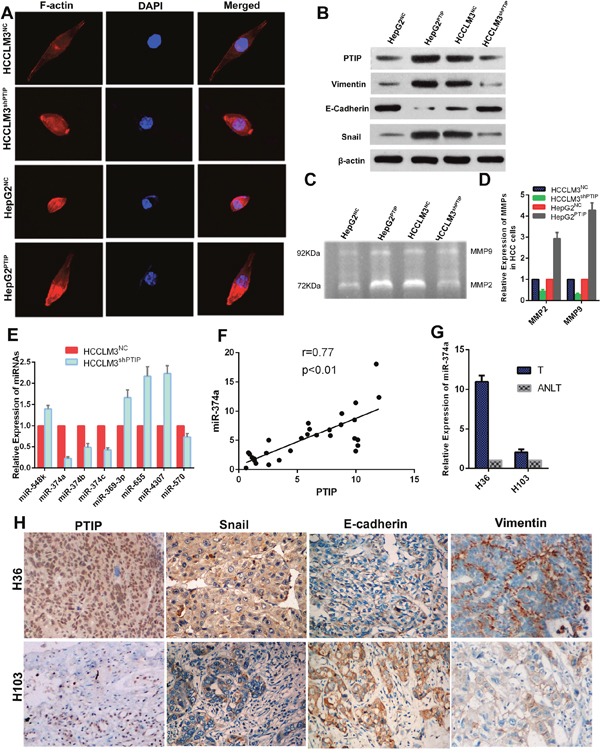
PTIP induces epithelial-mesenchymal transition (EMT) through miR-374a in hepatocellular carcinoma **(A)** Representative images of cytoskeleton. **(B)** Western blot analyzed PTIP and EMT markers expression in HCC cells with PTIP overexpression or knockdown, and their control cells. **(C)** PTIP enhanced metalloproteinase (MMP-2 and MMP-9) expression in HCC cells through gelatin zymography assay. **(D)** The expression of MMP-2 and MMP-9 was examined by qRT-PCR. The results showed that PTIP promoted the expression of MMP-2 and MMP-9. **(E)** The potential microRNA target of PTIP was detected by quantitative real-time PCR (qRT-PCR). The results showed that the expression of miR-374a was significantly lower in HCCLM3^shPTIP^ cells. **(F)** miR-374a was detected in thirty pairs of HCC tissues and corresponding ANLT by qRT-PCR. Correlation analysis showed that PTIP was positively related with miR-374a (r=0.77, P<0.01). **(G)** Referred to the previous results of PTIP expression in human tissues, a sample (H103) with low PTIP expression and a sample (H36) with high PTIP expression were chosen. **(H)** Expression of PTIP, Snail, E-cadherin and vimentin were examined by IHC. PTIP, Snail and Vimentin showed positive correlation with miR-374a;E-cadherin showed negative correlation with miR-374a.

Cellular morphological change in PTIP-expressing cells was analyzed through F-actin immunofluorescence staining. The results showed that PTIP overexpression in HepG2 cells induced the reorganization of actin cytoskeleton, while down-regulation of PTIP in HCCLM3 changed the cell appearance to more cobble-stone morphology (Figure 5A). These data indicated that PTIP might be involved in the epithelial-mesenchymal transition. Considering the function of PTIP and morphological change in HCC, we speculated that PTIP might promote HCC metastasis by facilitating EMT. To confirm this, the expression levels of EMT markers, E-cadherin, Vimentin and Snail were detected. The results showed that ectopic expression of PTIP in HepG2 cells increased the mesenchymal markers, Vimentin and Snail, and decreased the expression of the epithelial marker, E-cadherin. Whereas the knockdown of PTIP in HCCLM3 cells increased E-cadherin expression, decreased Vimentin and Snail expression (Figure 5B). The invasion ability of PTIP in HCC cells was tested using the getatin zymography and qRT-PCR. MMP-2 and MMP-9 expression decreased in the HCCLM3^shPTIP^ cells compared with the control cells, while MMP-2 and MMP-9 expression increased in PTIP ectopic expression HepG2 cells compared with the control cells (Figure 5C, 5D). These results showed that PTIP promoted HCC progression by facilitating EMT.

PTIP was originally identified as Pax2 transactivation domain interaction protein in the yeast two-hybrid system [5]. Previous studies showed PTIP could regulate the transcription of multiple genes [10, 13], but no research focused on PTIP regulating miRNA expression. To reveal how PTIP affected HCC metastasis, we attempted to identify the miRNA targets that were regulated by PTIP. Three databases, including TargetScan, PicTar, and miRanda, were searched for the candidate hits. We selected 8 potential microRNAs including miR-548k, miR-374b, miR-374a, miR-369-3p, miR-374c, miR-655, miR-4307, miR-570. Then, we investigated the expression of the above-mentioned eight potential targets through qRT-PCR in HCCLM3^shPTIP^ and HCCLM3^NC^ cells. We found that PTIP knockdown decreased the expression of miR-374a by about 80%, indicating miR-374a might be as a target of PTIP (Figure 5E). Then, we detected miR-374a in thirty pairs of HCC tissues and ANLT by qRT-PCR. Correlation analysis showed that PTIP was positively related with miR-374a(r=0.77, p<0.01) (Figure 5F). To confirm this result, qRT-PCR and IHC were performed to investigate the expression of PTIP, miR-374a, Snail, Vimentin, and E-cadherin in HCC with high PTIP expression (H36) and low PTIP expression (H103). The results showed that miR-374a, Snail and Vimentin had positive correlation with PTIP expression; however, E-cadherin had negative correlation with PTIP (Figure 5G, 5H). Taken together, these data showed that PTIP might promote EMT by facilitating miR-374a.

## DISCUSSION

Recurrence and metastasis are responsible for the most common lethal outcomes after curative resection in HCC [3, 4]. It is critical to gain a better understanding of the mechanism underlying HCC metastasis. PTIP promotes the transcription of multiple genes by recruiting the MLL3/MLL4 methyltransferase complex to gene-specific promoters or enhancers [9, 10, 14]. PTIP plays important roles in maintaining the embryonic stem cell pluripotency, and is involved in vasculogenesis and angiogenesis during embryogenesis [6, 7]. However, the clinical significance and biological function of PTIP in HCC remains unclear. In this study, we demonstrated for the first time that PTIP mRNA and protein level were prominently up-regulation in HCC tissues and liver cancer cell lines. We also found that high PTIP expression was associated with poor clinicopathological characteristics including liver cirrhosis. The results of Kaplan-Maier analysis showed that PTIP was an independent risk factor of OS and DFS. Interestingly, high PTIP expression with cirrhotic had significantly lower OS and DFS time. These results showed that PTIP might be a crucial factor in the metastasis of HCC. PTIP is a nuclear-protein, not a secretory protein [5], PTIP protein did not detected in serum. In order to avoid tumor implantation metastasis, biopsy is seldomly performed in patients who are suspected to have HCC. For this reason, PTIP is likely to be used as a prognostic indicator for HCC after hepatectomy, if large sample analysis confirmed this correlation in future studies.

To gain insight into the functional role of PTIP in HCC, we overexpressed PTIP in HepG2 cells and knocked down PTIP in HCCLM3 cells. The results showed that the knockdown of PTIP in HCCLM3 cells markedly decreased cell proliferation, migration and invasion, while overexpression of PTIP in HepG2 promoted cell proliferation, migration and invasion. Similarly, we confirmed that PTIP promoted tumor formation and metastasis by using animal model. These results supported that PTIP facilitated the invasion and metastasis of HCC *in vitro* and *in vivo*.

It has been reported that PTIP can promote the transcription of multiple genes dependently or independently from the associated MLL3/MLL4 methyltransferase complex [9, 11, 13, 15, 16]. Deficiency in PTIP protects replication forks from degradation and rescues the lethality of Brca2 knockout embryonic stem cells [12]. High PTIP expression was associated with increased progression-free survival [12]. But we found that high PTIP expression predicted poor prognosis. It is likely that PTIP might promote HCC metastasis by other mechanism. Our bioinformatical analysis identified eight potential microRNAs that might be regulated by PTIP, including miR-548k, miR-374b, miR-374a, miR-369-3p, miR-374c, miR-655, miR-4307, miR-570. qRT-PCR in HCCLM3^shPTIP^ and HCCLM3^NC^ cells showed miR-374a might be a potential target regulated by PTIP. MiR-374a has been reported to be involved in the progress of various types of cancer including HCC patients [17–21]. It was upregulated in HBV-HCC tissue and significantly correlated with prognosis-related clinical features, and it could promote HCC cells proliferation, migration and invasion [20, 22]. MiR-374a also activates Wnt/β-catenin signaling to

promote EMT and metastasis in breast cancer and HCC [20, 23]. The qRT-PCR results showed that miR-548k, miR-369-3p, miR-655 and miR-4307 increased in PTIP knockdown HCC cells. It has been reported that miR-655 as an EMT-suppressive microRNA suppressed invasion of tumors [24, 25]. PTIP might suppress the expression of miR-655. This needs further study in the future. Until now, miR-548k, miR-369-3p and miR-4307 have not been reported in tumors. miR-374a, miR-374b, miR-374c and miR-570 decreased in HCCLM3shPTIP cells. The change of miR-570 was not obvious. miR-374b has been reported to promote progression in gastric cancer [26]. While it was negative with breast cancer and T-cell Lymphoblastic Lymphoma [27, 28]. miR-374c was downregulated in Merkel cell carcinoma [29]. Therefore, it is highly likely that PTIP promotes the invasion and metastasis of HCC through regulating EMT by miR-374a. Then, we detected miR-374a in thirty pairs of HCC tissues and ANLT by qRT-PCR. Correlation analysis indicated that PTIP was positive related with miR-374a. Furthermore, we found that the low expression level of PTIP was associated with low miR-374a expression and low expression of mesenchymal markers (Vimentin and Snail). In contrast, the epithelial marker E-cadherin expression was the opposite. Meanwhile, PTIP increased MMP-2 and MMP-9 expression in HCC cells. Taken together, our result demonstrated that PTIP might promote the invasion and metastasis of HCC via EMT by miR-374a.

In conclusion, PTIP is overexpressed in HCC and is associated with poor prognosis of HCC patients. Our study demonstrates, for the first time, that PTIP plays a vital role in HCC invasion and metastasis *in vitro* and *in vivo*. Furthermore, we also show that PTIP might promote HCC progression via EMT through miR-374a. PTIP may be developed as a valuable prognostic biomarker and a potential therapeutic target in HCC.

## MATERIALS AND METHODS

### Patient specimens

A total of 195 HCC specimens were recruited from 2002 to 2012. The medical centers involved are the Affiliated Hospital of Chengdu University, Xiangya Hospital of Central South University and Chongqing Dazu District People's Hospital. All patients in our study did not receive any neoadjuvant chemotherapy or local ablation before hepatectomy. There were 21 patients who did not obtain R0-resected. 136 patients were HBV related HCC. Hepatitis C infected patients and alcohol consumers were excluded. Among these patients, 30 matched HCC and the adjacent non-tumor liver tissue (ANLT) specimens were collected for quantitative real-time RT–PCR (qRT-PCR). Paraffin-embedded specimens were randomly collected for immunohistochemistry. Histopathology was verified by two independent histopathologists. Serum samples from 10 HCC patients were used for ELISA assays of PTIP. Prior informed consent was obtained from all patients and the study protocol was approved by the Ethics Committee of Affiliated Hospital of Chengdu University.

### Follow-up and prognostic study

All of the patients were regularly followed-up by the same experienced team. The latest follow-up was terminated on March, 2016. Surveillance for the tumor recurrence and metastasis was done by monitoring of alpha-fetoprotein (AFP) levels and ultrasonography every 3 month. If recurrence or metastasis was suspected, computed tomography (CT) scan and/or magnetic resonance imaging (MRI) were performed. Overall survival (OS) was defined as the time from hepatectomy to death or to the date of the last follow-up. Disease-free survival (DFS) was defined as the time from hepatic resection to the date when recurrence or metastasis was detected. Time to recurrence (TTR) [30, 31] was defined as the interval between the date of surgery and the date of any diagnosed relapse (intrahepatic recurrence and extrahepatic metastasis). The complete clinical and pathological features of these patients are listed in Table 1 in detail.

### Cell lines

SMMC7721 and HCCLM3 cell lines were purchased from Shanghai Institute of Cell Biology, Liver Cancer Institute of Fudan University. L02, HepG2, Hep3B and Huh7 were purchased from American Type Culture Collection (ATCC Rockville, MA). Above cell lines were cultured in high glucose DMEM supplemented with 10% fetal bovine serum, 100 U/mL penicillin, and 100 mg/mL streptomycin at 37° in a humidified incubator under 5% CO_2_.

### RNA isolation and quantitative real-time RT–PCR

Extraction of the total RNAs from HCC tissues and cells was carried out according to the MiRCURY RNA Kit (Exiqon, Denmark) instructions. Real-time PCR was performed using the SYBR Green Real-time PCR Master Mix (TaKaRa Bio, Shiga, Japan) as described. GAPDH was used as an internal control for mRNA. RNU6B (U6) was measured as an internal control for miRNA. All of the primers were synthesized by Sangon (Shanghai, China). The primer sequences tested in this study were listed in Supplementary Table 1. All qRT-PCRs were performed in triplicates.

### Western blot analysis

Total proteins were extracted with RIPA lysis buffer and separated by 10% sodium dodecyl sulfate polyacrylamide gel electrophoresis (SDS-PAGE) and then transferred onto polyvinylidene fluoride membranes (Millipore, Bedford, Mass). The membrane was blocked with 5% skimmed milk and incubated with the appropriate antibody. Then, enhanced chemiluminescence regents (Thermo Scientific, Waltham, MA) were used to detect the antigen-antibody complex. The PTIP antibody was purchased from Abcam (Massachusetts, US). Antibodies for Snail, E-cadherin, Vimentin, β-action and corresponding secondary antibodies were obtained from Santa Cruz Biotechnology (Santa Cruz, CA).

### Immunohistochemical analyses

The paraffin-embedded tissue sections were cut into 4μm thickness. Immunohistochemical staining for PTIP, Snail, Vimentin, E-cadherin, and N-cadherin were performed using the polymer HRP detection system (Zhongshan Goldenbridge Biotechnology). The expression levels of PTIP were scored according to the staining intensity and the percentage of positively stained tumor cells. With 0 denotes ≤5% positive, 1 denotes 5 – 30%, 2 denotes 31 – 50%, 3 denotes 51 – 80% and 4 denotes >80% positive. PTIP protein expression was divided into a low expression group (0 - 1) and a high expression group (2 - 4) for further analysis.

### ELISA analysis

Levels of PTIP protein in serum samples from HCC patients was detected by ELISA kits (R&D, Minneapolis, MN, USA). A 96-well microplate was coated with 100μl of 5μg/ml PTIP antibody and incubated at 4 °C overnight. Serum samples were diluted (1:50) in sample diluents respectively before the assay. Tests were performed in accordance to manufacturer's instructions.

### Establishment of PTIP knockdown and overexpression cells

PTIP knockdown and ectopic expression lentivirus as well as the relative negative control(NC) lentivirus were purchased from GeneChem (Shanghai, China). The three candidate hairpin sequences were listed in Supplementary Table 2. PTIP overexpression CDS area address: http://www.ncbi.nlm.nih.gov/nuccore/NM_007349.3. Full-length human PTIP ectopic expression lentivirus was transfected into HepG2 cells, and lentiviral containing short hairpin RNAs (shRNA) targeting PTIP was transfected into HCCLM3 cells according to the manufacturer's instructions. 1×10^5^ cells were infected with 1×10^8^ lentivirus in the presence of 1ul polybrene (GeneChem, Shanghai, China). Cells transfected with empty vector were used as controls. In this study, the infection efficiency of lentivirus was over 90%. Stable transfection lentivirus cell lines were used for subsequent assays.

### Immunofluorescence

F-actin immunofluorescence staining was used to analyze the cell skeleton. Stably transfected cells grown on coverslips were fixed, and then incubated with rhodamine-conjugated phalloidin (Beyotime Institue of Biotechnology, Jiangsu, China). The samples were observed and analyzed with an inverted microscope TE-2000S (Nikon, Tokyo, Japan).

### MTT assay and colony formation assay

For MTT assay, HCC Stably infected cells were seeded into 96-well plates at a density of 5×10^3^ cells per well. Six wells of each group were detected every day. Cells were treated with 100μl fresh medium containing MTT 0.5 mg/ml (Sigma, USA), and incubated at 37° for 4 hours, then the cells were dissolved in 100μl of DMSO and shaken at room temperature for 10 mins. The absorbance was measured at 570 nm. Experiments were performed in triplicate. For colony formation assays, HCC cells were seeded into 35mm dishes (Corning Costar Corp, Corning, NY) at a density of 5×10^2^ cells per well. Then, the cells were incubated at 37°C, 5% CO2 for 2 weeks. Subsequently, the cells were stained with crystal violet (Bogoo, Shanghai, China). We captured the dishes with a camera (Nikon, Tokyo, Japan). The colonies in the dishes were counted and compared.

### Wound healing and transwell assay *in vitro*

Wound healing assay and Transwell invasion assay were used to assess the ability of cell migration. For the wound healing assay, appropriate number of HCC cells were seeded into 55-mm dishes and cultured for one day. When the cells were closed to 100% confluence, a scratch line was created with a 100ul pipette tip. And then, the cells were cultured for another 24 hours, the rate of closure was assessed through an inverted microscope TE-2000S (Nikon). For the Transwell invasion assay, about 1×10^5^ cells were seeded into the upper chamber of the insert with Matrigel coated membrane (BD Biosciences, Franklin Lakes, NJ). After 24 hours, the cells and gel in the upper chamber were removed carefully and cells adhering to the underside of the membrane were stained with 0.1% crystal violet (Bogoo, Shanghai, China) and 20% methanol. The numbers of cells was counted under an inverted microscope (Nikon). For each experimental group, the assay was performed in triplicate.

### Gelatin zymography assay

To evaluate the invasive ability of PTIP in HCC cells, gelatin zymography was carried out. HCC cells were incubated in DMEM for 24 hours. The supernatants were collected. Then, the samples were separated by electrophoresis on a 10 % SDS-PAGE gel electrophoresis containing 0.1 % gelatin (Sigma-Aldrich). After electrophoresis, the gel was stained with Coomassie Brilliant Blue R-250 for 30min at room temperature. The presence of gelatinolytic activity was identified as clear bands on a uniform blue background. Molecular weight was estimated by reference to prestained SDS-PAGE markers.

### Tumor formation and metastasis assays *in vivo*

The HCC metastatic mouse model was constructed by using HCCLM3^shPTIP^, HepG2^PTIP^ or control cells. Cells (2 × 10^6^) were injected subcutaneously into the left upper flank regions of nude mouse (3-4 weeks of age, male, BALB/c). After one month, the subcutaneous tumor tissues were obtained and cut into commensurate fragments of approximately 1 mm^3^. Then, each fragment was implanted into the liver (four mice for each group). After 6 weeks of the implantation, the mice were sacrificed, and the tumors volume (*V*) was calculated as follows: *V*= 1/2(L × W^2^). Livers and lungs were harvested and fixed with phosphate-buffered neutral formalin. Serial sections were subjected to histopathological analysis by hematoxylin and eosin (H&E) staining, the metastatic foci was recorded by specialized pathologists. Then, the rate of tumors metastasis was compared between the groups of each panel. The mice were housed and manipulated according to the protocols and approved by the Medical Experimental Animal Care Commission of Affiliated Hospital of Chengdu University.

### Statistical analysis

All data were analyzed by SPSS 21.0 software. Data were presented as the mean ±SEM from at least three independent experiments. The differences between two groups were analyzed by Student's t test and the Pearson χ^2^ was used for the analysis of variables data. Overall survival and disease-free survival curves were obtained using the Kaplan-Meier method, and differences in survival between the high PTIP expression group and low expression group were evaluated using the log-rank test. Univariate and multivariate analysis were analyzed with Cox proportional hazards regression model to verify the independent risk factors. *P* value< 0.05 was considered to be statistically significant.

## SUPPLEMENTARY MATERIALS FIGURES AND TABLES


