# Supplementary material for: PTIP promotes recurrence and metastasis of hepatocellular carcinoma by regulating epithelial-mesenchymal transition
Source: Oncotarget. 2017 Mar 22;8(35):58184–98. doi: 10.18632/oncotarget.16436 (PMC5601643; doi:10.18632/oncotarget.16436)
Supplement: Supplementary file 1 [file oncotarget-08-58184-s001.pdf]

PTIP promotes recurrence and metastasis of hepatocellular carcinoma by regulating epithelial-mesenchymal transition

SUPPLEMENTARY FIGURES AND TABLES

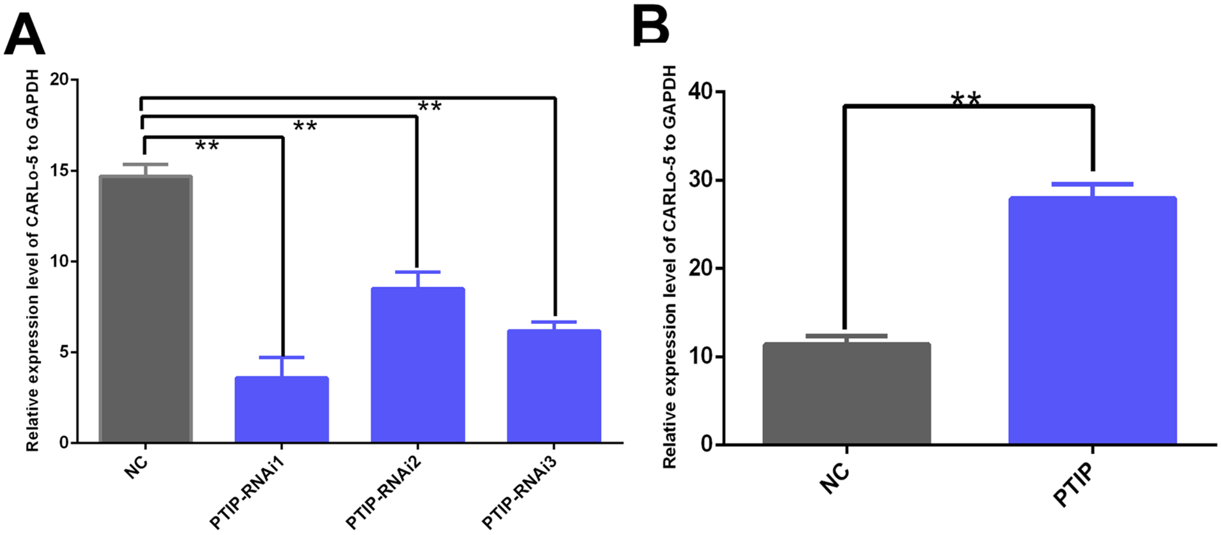

Supplementary Figure 1: The efficiency of PTIP knockdown and ectopic expression in HCC cells. **(A)** HCCLM3 cells were treated with three siRNAs targeting PTIP and Negative control. The most efficiency of PTIP knockdown was PTIP-RNAi1. **(B)** HepG2 cells were transfected with PTIP overexpression lentivirus. Efficiency of PTIP overexpression was more than two fold.

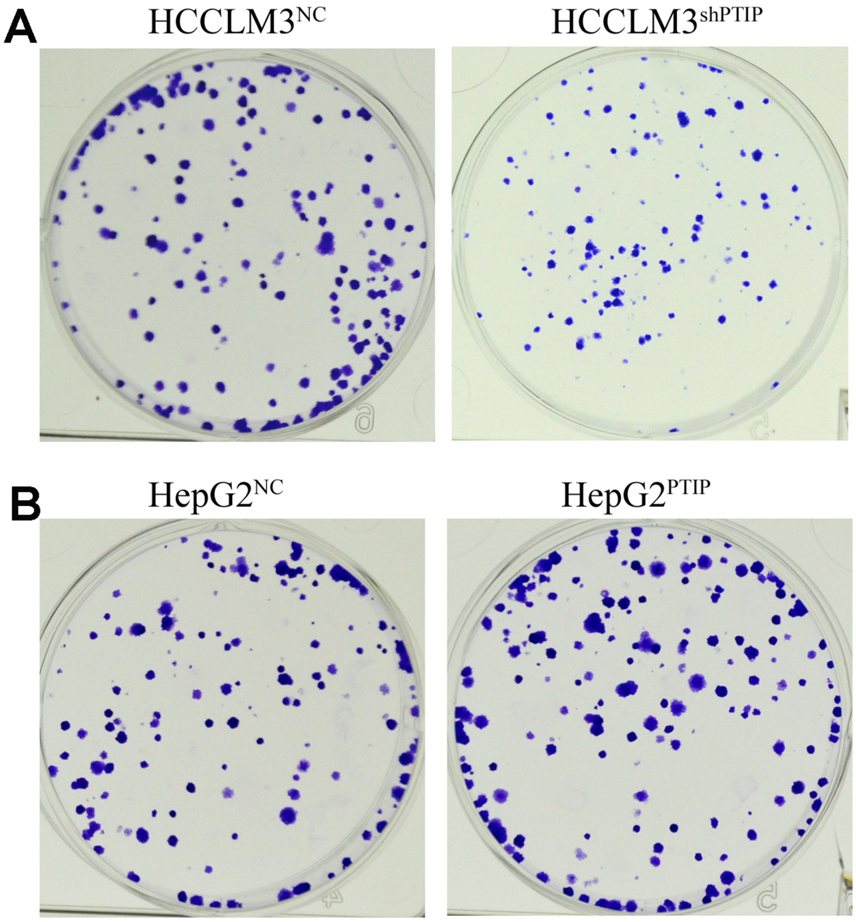

Supplementary Figure 2: PTIP promoted proliferation HCC cells *in vitro*. Cell growth of HCC cells was examined with colony formation assays. **(A)** The results showed that HCCLM3^{shPTIP} grew slower than HCCLM3^{NC}. **(B)** HepG2^{PTIP} grew faster than HepG2^{NC}, Original magnification ×400. NC: Negative Control.

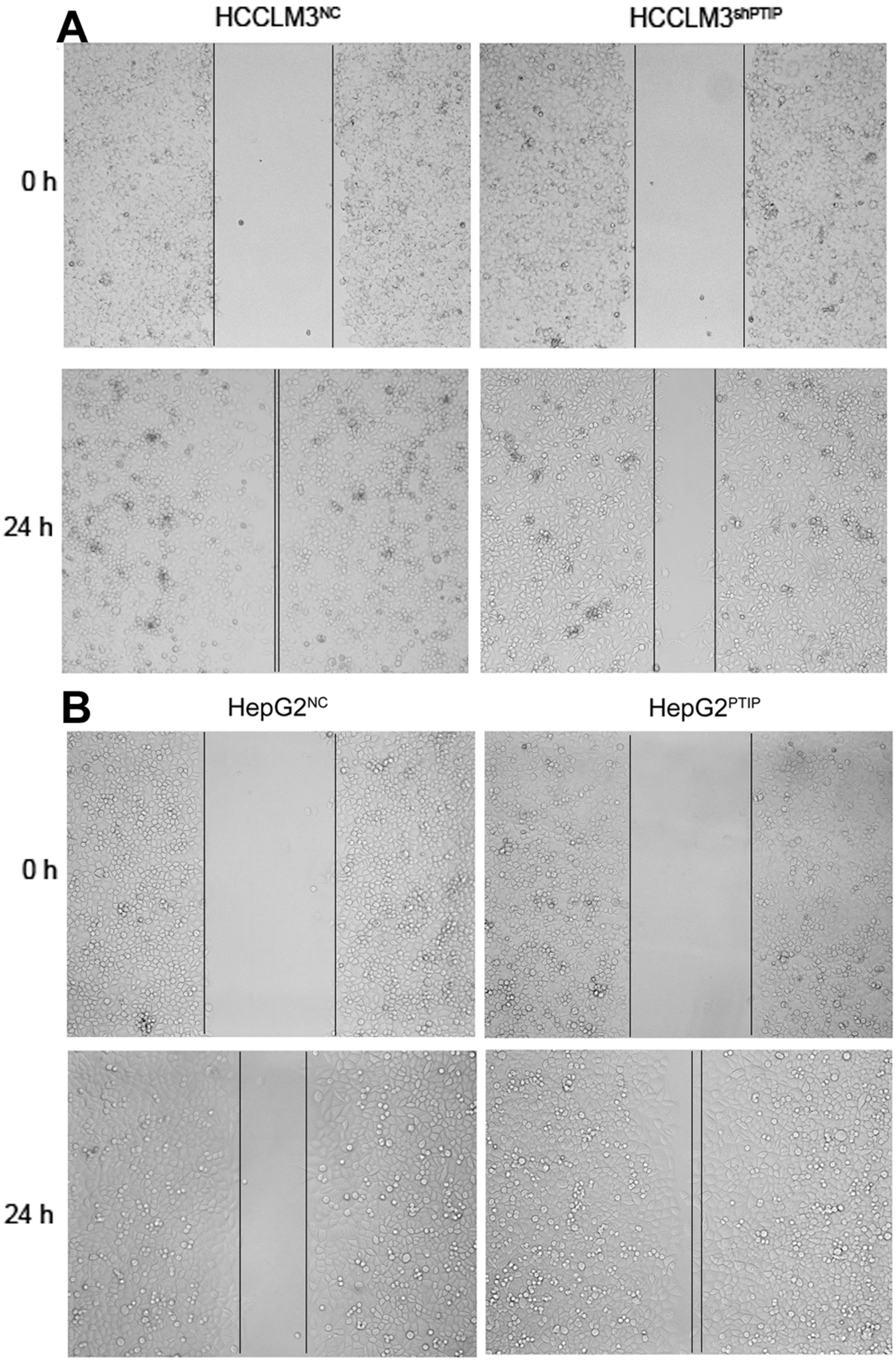

Supplementary Figure 3: PTIP significantly promoted migration of HCC cells *in vitro*. Migration of HCC cells were measured with wound healing assays. **(A)** HCCLM3^{shPTIP} cells closed much slower than HCCLM3^{NC}. **(B)** The closure of HepG2^{PTIP} was more than HepG2^{NC}. Original magnification ×400. NC: Negative Control.

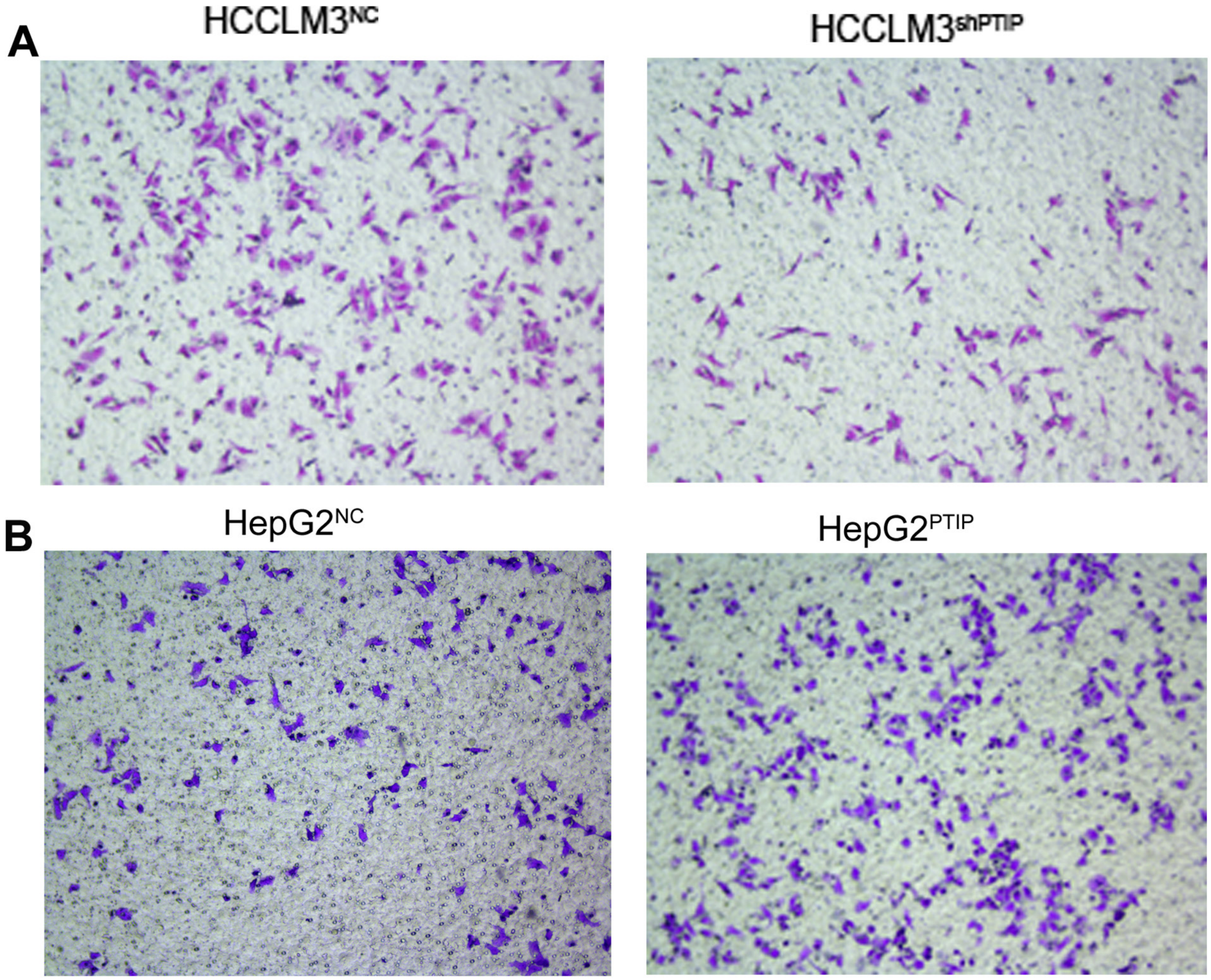

Supplementary Figure 4: PTIP significantly promoted invasion of HCC cells *in vitro*. Invasion of HCC cells were measured with transwell assays. **(A)** HCCLM3^{shPTIP} cells passed through the matrigel were significantly less than that of HCCLM3^{NC}; **(B)** HepG2^{PTIP} cells passed through the matrigel was significantly more than that of HepG2^{NC}. Original magnification ×400. NC: Negative Control.

Supplementary Table 1: List of the primer used in this study

Name[a]		Sequence(5'→3')
PTIP	F	GCAGCAGCAGCAGCTTTTTG
	R	TGCTCGGGATAGTCCGCAAT
miR-374a	F	CGCGATAGCGGCGGTTATAATAC
	R	ATCCAGTGCAGGGTCCGAGG
miR-374b	F	CGCGCGGATATAATACAACCTGC
	R	ATCCAGTGCAGGGTCCGAGG
miR-374c	F	CCGGCGGATAATACAACCTGCTA
	R	ATCCAGTGCAGGGTCCGAGG
miR-548k	F	CGCCGGAAAAGTACTTGCGGAT
	R	ATCCAGTGCAGGGTCCGAGG
miR-369-3p	F	CGCGGCGCAATAATACATGGTTG
	R	ATCCAGTGCAGGGTCCGAGG
miR-655	F	CGCCGCGGATAATACATGGTTAAC
	R	ATCCAGTGCAGGGTCCGAGG
miR-4307	F	CGCGCGCGAGAATGTTTTTTCCT
	R	ATCCAGTGCAGGGTCCGAGG
miR-570	F	CGCGCGACGAAAACAGCAATTAC
	R	ATCCAGTGCAGGGTCCGAGG
MMP2	F	CAGCCAACTACGATGATGA
	R	GTGCCAAGGTCAATGTCA
MMP9	F	TGACAGCGACAAGAAGTG
	R	CAGTGAAGCGGTACATAGG
GAPDH	F	AACGGATTTGGTCGTATTGG
	R	TTGATTTTGGAGGGATCTCG
U6	Sense	CTCGCTTCGGCAGCACATATA

[a] F, forward primer; R, reverse primer.

Supplementary Table 2: List of the three candidate hairpin sequences of PTIP used in this study

Sequences 1	sense	5′-CTCCACACAGGCATAGAGTGTCTGCT-3′
	antisense	5′-CACCATCCACATACAAGAATACATGCTCCA-3′
Sequences 2	sense	5′-CGCCCCCGAGGCCCACCGACTCTAGA-3′
	antisense	5′-CACCATCCACATACAAGAATACATGCTCCA-3′
Sequences 3	sense	5′-TTCTGCTCGGCGGGCAGCGGGCGGATCGAT-3′
	antisense	5′-GAACGTCTGACCGCCCATAAAAATGCCGTG-3′
